# Aphid Thermal Tolerance Is Governed by a Point Mutation in Bacterial Symbionts 

**DOI:** 10.1371/journal.pbio.0050096

**Published:** 2007-04-10

**Authors:** Helen E Dunbar, Alex C. C Wilson, Nicole R Ferguson, Nancy A Moran

**Affiliations:** Department of Ecology and Evolutionary Biology, University of Arizona, Tucson, Arizona, United States of America; Oxford University, United Kingdom

## Abstract

Symbiosis is a ubiquitous phenomenon generating biological complexity, affecting adaptation, and expanding ecological capabilities. However, symbionts, which can be subject to genetic limitations such as clonality and genomic degradation, also impose constraints on hosts. A model of obligate symbiosis is that between aphids and the bacterium *Buchnera aphidicola,* which supplies essential nutrients. We report a mutation in *Buchnera* of the aphid Acyrthosiphon pisum that recurs in laboratory lines and occurs in field populations. This single nucleotide deletion affects a homopolymeric run within the heat-shock transcriptional promoter for *ibpA,* encoding a small heat-shock protein. This *Buchnera* mutation virtually eliminates the transcriptional response of *ibpA* to heat stress and lowers its expression even at cool or moderate temperatures. Furthermore, this symbiont mutation dramatically affects host fitness in a manner dependent on thermal environment. Following a short heat exposure as juveniles, aphids bearing short-allele symbionts produced few or no progeny and contained almost no *Buchnera,* in contrast to aphids bearing symbionts without the deletion. Conversely, under constant cool conditions, aphids containing symbionts with the short allele reproduced earlier and maintained higher reproductive rates. The short allele has appreciable frequencies in field populations (up to 20%), further supporting the view that lowering of *ibpA* expression improves host fitness under some conditions. This recurring *Buchnera* mutation governs thermal tolerance of aphid hosts. Other cases in which symbiont microevolution has a major effect on host ecological tolerance are likely to be widespread because of the high mutation rates of symbiotic bacteria and their crucial roles in host metabolism and development.

## Author Summary

Aphids are sap-feeding insects that depend on obligate bacterial symbionts of the genus *Buchnera* for biosynthesis of needed nutrients. Studying *Buchnera* gene expression in pea aphids, we identified a recurring mutation (a single-base deletion) in the transcriptional promoter of the small heat-shock protein, *ibpA*. This mutation arose and was fixed twice in sublines derived from a single female aphid in the lab and kept at constant 20 °C. Experiments using aphid lines that differed only in the presence of this *Buchnera* mutation revealed that it eliminates the *ibpA* transcriptional response to heat shock and affects *ibpA* expression at low temperatures. In aphids containing *Buchnera* with the mutation, a short heat treatment as juveniles leads to elimination of most or all symbionts and to reproductive failure; the same treatment has little effect on aphids containing *Buchnera* without the mutation. Conversely, at constant lower temperatures, aphids with *Buchnera* bearing this mutation enjoy a reproductive advantage. Pea aphid populations are polymorphic for the *Buchnera* mutation, suggesting that it is maintained at substantial frequencies by selection. This study indicates that mutations in obligate symbionts can have major consequences for host fitness and geographic distributions.

## Abstract 3

A recurring single-nucleotide deletion in a heat-shock transcriptional promoter in the bacterial*Buchnera* symbiont of the pea aphid*(Acyrthosiphon pisum)* dramatically affects host fitness in a thermal-dependent manner.

## Introduction

A model for a heritable, mutually obligate symbiosis is that between aphids and the bacterial symbiont, *Buchnera aphidicola,* which provisions hosts with essential amino acids that are rare or absent from their phloem sap diet [1,2]. Although this intimate mutualism has been critical in enabling aphids to exploit the phloem sap-feeding niche and to diversify onto many plant groups, aphids are constrained by *Buchnera*'s ecological tolerances. These constraints are potentially severe because *Buchnera* genomes are highly reduced and show no incidence of recombination or gene acquisition [3], reflecting strict clonality and maternal transmission for over 100 million years [4]. In particular, aphid ability to colonize geographic regions subject to high temperatures appears to be limited in part by dependence on *Buchnera*. Aphids can be rendered infertile by heat that kills *Buchnera* cells [5]. In field populations of Acyrthosiphon pisum (pea aphid), temperatures of 25–30 °C depress *Buchnera* densities within hosts [6].

A major impact of heat on cellular function stems from degradation of protein secondary structures. Most cellular organisms have the capacity to respond to heat stress by directing transcription and/or translation to the production of chaperones and proteases that deter protein aggregation [7]. In *Buchnera* of another aphid species *(Schizaphis graminum),* a study using a full-genome microarray showed that the symbiont heat-shock response is restricted to four transcriptional promoters, affecting expression of five heat-shock genes [8]; the same set of heat-shock promoters and genes are found in the genome of *Buchnera* of A. pisum [1,9]. Additionally, numerous genes normally up-regulated in response to heat are constitutively highly expressed in *Buchnera,* a feature interpreted as an adaptation to maintain function of proteins destabilized by mutational accumulation [8].

In this report, we describe the discovery of a single-base regulatory mutation in *Buchnera* that reduces the transcriptional response to heat stress of *ibpA,* a universal small heat-shock gene. This mutation, which was fixed twice in laboratory lines maintained under cool conditions and which is present in *Buchnera* in field populations of *A. pisum,* diminishes the ability of hosts to withstand thermal stress, but improves reproduction under cool conditions.

## Results

### A Single-Base Deletion Nearly Eliminates Heat-Shock Response of *ibpA*

**Figure 1:**
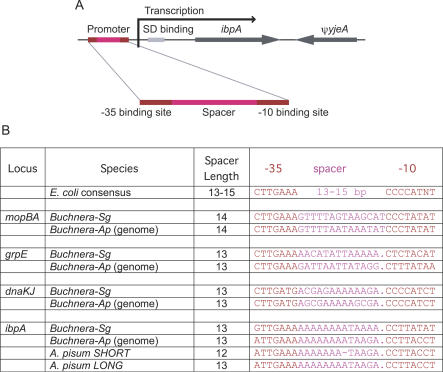
Heat-Shock Promoter Governing Expression of the Heat-Shock Gene *ibpA* in *Buchnera* (A) Schematic of regulation of *ibpA* expression showing position of heat-shock promoter. (B) Sequence of E. coli consensus heat-shock promoter and of all heat-shock promoters of *Buchnera* of S. graminum and of A. pisum. Sequences from published complete genome data were confirmed for both species. The only variant is the *ibpA* promoter allele, which has a shorter spacer length, resulting in almost complete loss of the heat-shock response of *ibpA*.

**Figure 2:**
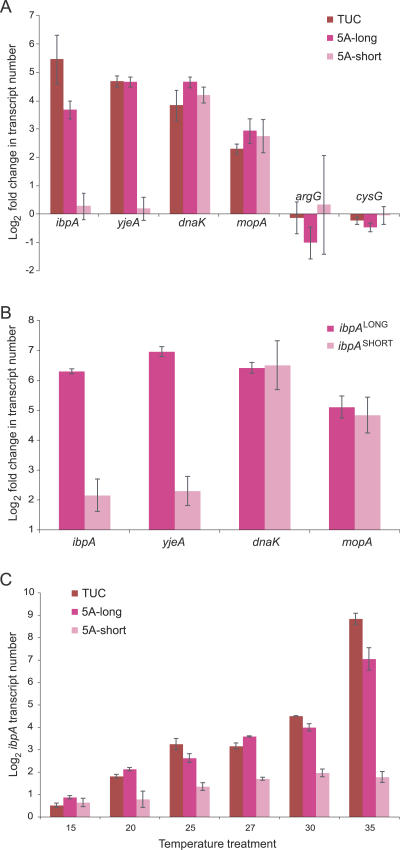
Response to Heat Treatment in *Buchnera*-Ap lines, for Genes Typically Up-Regulated in Response to Heat (A) Gene expression changes in response to heat treatment (35 °C) as measured from a microarray experiment, including two control genes *(argG* and *cysG)* that did not show significant responses to heat treatment. Each bar represents the mean of technical and biological replicates of each line (four arrays/aphid line) ± standard error. (B) Gene expression changes in response to heat (35 °C) as measured with RT-qPCR using several A. pisum lines containing *Buchnera* with either short or long *ibpA* promoter alleles. For the long allele, *n* = 4 (three lines), and for the short allele, *n* = 2 (two lines). Error bars indicate means ± standard errors. (C) Effects of exposure to different temperatures on *ibpA* expression for three aphid lines bearing *Buchnera* with different *ibpA* promoter alleles. The two 5A lines differ only in the single-base deletion affecting the length of the *ibpA* spacer; whereas TUC matches 5A-long for the *ibpA* promoter sequence, but shows other differences from 5A lines throughout the *Buchnera* genome. Pairwise differences between the two 5A-derived lines are significant at every temperature, with the long allele always showing higher expression relative to a control gene *(cysG)*. TUC has other sequence differences, and these appear to have minor effects on *ibpA* expression.

**Figure 3:**
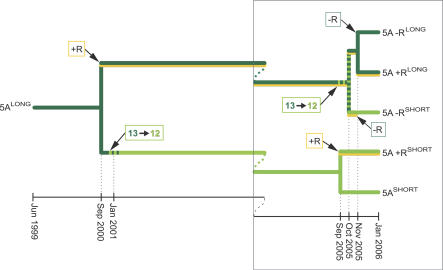
Chronology of *ibpA* Heat-Shock Promoter Evolution in Sublines of the 5A Line Line 5A was initiated from a single female in June 1999. In September 2000, it was transfected with a secondary symbiont to generate 5A^+^R^LONG^. Subsequently the 5A line acquired the first mutation to the short promoter allele, which became fixed. An identical mutation rose to near fixation in the 5A^+^R^LONG^ culture in October 2005; both alleles were isolated from that colony to yield 5A^+^R^SHORT^ and 5A^+^R^LONG^. Line 5A^+^R^SHORT^ lost its *Candidatus* Serratia symbiotica to generate line 5A^−^R^SHORT^, and then in November 2005, 5A^+^R^LONG^ was cured of *Candidatus* Serratia symbiotica to generate line 5A^−^R^LONG^. Thus the mutation occurred and rose to fixation or near fixation twice in sublines of the 5A laboratory line (Figure 1B).

Using a microarray bearing double-stranded probes for a partial set of genes of both A. pisum and its *Buchnera* [9], we identified two *Buchnera* probes for which different A. pisum lines showed large differences in response to heat treatment (4 h at 35 °C versus control conditions of constant 20 °C). These probes corresponded to *ibpA,* which encodes a small heat-shock protein present in almost all cellular organisms, and *ψyjeA,* a pseudogene positioned immediately downstream of *ibpA* on the opposite strand (Figure 1A). The *Buchnera* of two aphid lines (TUC and 5AR) showed highly significant up-regulation of *ibpA* and *ψyjeA* following heat treatment (25–42-fold increase, *p* < 7.26 × 10^−7^, and 13–25-fold increase, *p* < 2.14 × 10^−11^, respectively). In contrast, another line (5A0) showed no significant up-regulation of the same genes (*ibpA, p* = 0.56, and *ψyjeA, p* = 0.65) (Figure 2A). No other genes showed substantial differences in response among these three aphid lines. Lines 5AR and 5A0 diverged only 5 y earlier, when they were divided into two sublines in the laboratory [10] (Figure 3). Increase in transcripts corresponding to *ψyjeA* was previously shown to result from transcriptional read-through from the *ibpA* promoter [8], so we hypothesized that the change in response of both genes was attributable to a single mutation that had occurred and become fixed during the previous 5 y following the separation of 5A0 and 5AR lines.

To determine the cause of the observed difference in response, we sequenced the relevant intergenic spacer in the *A. pisum* lines. The region upstream of *ibpA* in *Buchnera* of both A. pisum and of S. graminum contains a heat-shock promoter with high similarity to the consensus sequence of Escherichia coli (Figure 1) [1,3,8]. Heat-shock promoters are specific DNA sequences that bind the alternative sigma factor, σ32, which direct RNA polymerase to target genes under stress conditions. Our sequencing revealed that the σ32 promoter of line 5A0, the line lacking a strong *ibpA* transcriptional response in the microarray experiments, had a single-base deletion in the spacer between the −35 and −10 binding sites, whereas the promoter sequence of lines with a strong response (5AR and TUC) was identical to that of the previously sequenced genome for *Buchnera* of A. pisum (originating in Japan) (Figure 1B). This deletion involved the reduction of a mononucleotide run of 11 adenines to a run of ten adenines, with a corresponding reduction in the length of the overall spacer from 13 to 12 nucleotides. Thus, the spacer in 5AR, TUC, and the publicly available genome sequence is 13 base pairs (bp) long (“long,” Figure 1B), whereas the spacer in line 5A0 is 12 bp long (“short”).

By sequencing the promoter regions of other A. pisum lines (maintained in laboratory cultures) and of a series of preserved samples taken from these lines near the time of their establishment from field-collected individuals, we determined that the mutation from the long to the short allele arose and became fixed twice in the lab-reared cultures derived from line 5A. The first such mutation occurred in January 2001, and the same mutation recurred in October 2005 in a different line (Figure 3). The short allele was found in *Buchnera* of two additional laboratory lines, both of which had this allele at the time of establishment from independent field collections, as determined by screening samples taken soon after the lines were isolated from the field. The short allele was also found in symbionts of numerous field-collected individuals from Wisconsin and New York State, but was absent from collections from Arizona and Utah (Table 1, 13% of samples overall).

**Table 1:**
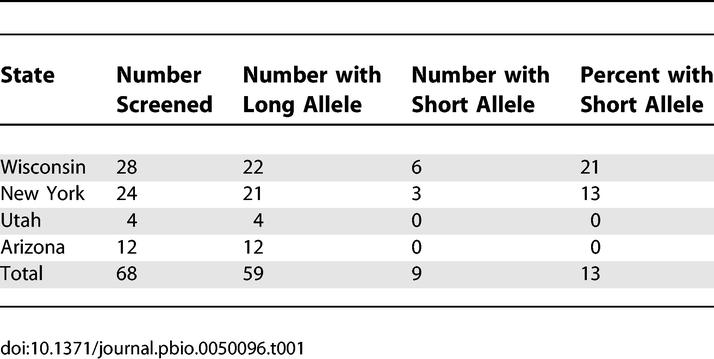
Frequency of *ibpA* Promoter Alleles in *Buchnera* of Field-Collected Pea Aphids from Localities in North America

To verify the effect of the polymorphism on gene expression, we used real-time reverse transcriptase quantitative PCR (RT-qPCR) to evaluate change in transcript abundance following heat stress for *ibpA* and *ψyjeA,* as well as two other heat-shock genes governed by other heat-shock promoters, for several A. pisum lines representing each of the two *Buchnera* alleles. Following heat treatment, every line containing *Buchnera* with the short allele showed little or no change in expression of *ibpA* and *ψyjeA,* and every line with the long-allele *Buchnera* showed dramatically increased expression of both *ibpA* and *ψyjeA,* with relatively minor difference among lines containing Buchnera with the same allele (Figure 2B). Furthermore, other heat-shock genes, *mopA* (= *groEL*) and *dnaK* (= *hsp70*), showed no expression differences between lines with the two *Buchnera* types (Figure 2B), indicating that the altered response was limited to *ibpA/ψyjeA* and that the global heat-shock response system was otherwise equivalent.

For the *Buchnera* of the two 5A lines (Figure 3), this single-base deletion in the *ibpA* spacer was the only sequence difference in the entire intergenic region upstream of *ibpA*. Furthermore, a genome-wide screen of all *Buchnera* sequence differences, using comparative genome hybridization to a tiling microarray, indicated that no other changes had occurred in the *Buchnera* genomes from these two lines (unpublished data). Therefore, the differences in *ibpA* expression among 5A lines can be confidently attributed to this deletion of a single adenine. For the other lines tested (from independent field collections), a G/T polymorphism occurred five bases upstream of the promoter-binding site (nine bases upstream of the poly-adenine tract). Long spacers occurred in genotypes with both the G and T residues. We conclude that the length of the spacer of the *ibpA* σ32 binding site has a large effect on *ibpA* expression following heat stress. Whereas most of the difference in expression is attributable to the spacer length, this G/T polymorphism could have some effect on *ibpA* expression, possibly resulting in the greater response in TUC than in the 5A lines with the long allele (Figure 2A).

In another experiment, we compared differences in *ibpA* expression across a wide range of temperatures, for three lines. Two lines were derived from 5A and differed only in the single-base deletion; the other (TUC) was from an independent collection and possessed the longer spacer as well as the G/T difference five bases upstream from the promoter binding site plus many other differences in the *Buchnera* genome (approximately 0.3% divergence; unpublished data). Again, severe heat stress (35 °C) resulted in much higher expression for both lines possessing long spacers (Figure 2C). At all temperatures, including cool ones (15 °C), the single-base deletion in the spacer resulted in lower expression of *ibpA,* based on significant differences in expression between 5A lines with and without the deletion (Figure 2C). TUC showed both significantly higher expression at 35 °C and significantly lower expression at 15 °C, as compared to either of the 5A lines, indicating that some other genomic differences are also affecting *ibpA* expression in a temperature-dependent manner. Nonetheless, the *ibpA* promoter spacer length has an overriding effect, at high temperatures, since all lines with the longer spacer showed dramatically higher response than lines with the short spacer (Figure 2A).

Our results on the effect of the single-base deletion on *ibpA* expression matches expectations based on mutational studies in *E. coli,* which has the same consensus heat-shock promoter (Figure 1B). In *E. coli,* a single-base change in the length of the σ32 promoter spacer has a major effect on the transcriptional response to heat stress [11]. The set of heat-shock promoters in E. coli includes spacer lengths of 13, 14, and 15 bp, with 14 showing the highest response. The shorter spacer, corresponding to a length of 12 bp, is thus expected to give little or no response, as we observed for *Buchnera* of *A. pisum.*

### Effect of *Buchnera* Mutation on Reproduction of Aphid Hosts Following Heat Stress

To determine the consequences of this *Buchnera* mutation for aphid fitness under different thermal regimes, we examined reproductive ability of aphids from different 5A-derived lines bearing *Buchnera* with the short and the long alleles for the *ibpA* heat-shock promoter, subjecting them to control conditions (20 °C constant temperature) and heat treatment (4 h of 35.5 °C on day 2 following birth). The latter treatment was designed to mimic the temperature changes experienced when an aphid falls from the plant onto the ground, where radiant heat can cause air in the few millimeters near the surface to be 5–25 °C above air temperatures near plant leaves (unpublished data). In an initial experiment, which included 5A-derived lines with both *Buchnera* alleles and with and without the facultative symbiont *Candidatus* Serratia symbiotica, no differences between aphids bearing *Buchnera* with the alternative alleles were evident at constant 20 °C (Wilcoxon tests for age at first reproduction: *p* = 0.14; 7-d fecundity: *p* = 0.11; progeny weight: *p* = 0.27; and maternal weight: *p* = 0.72). Power was low in these experiments due to small samples sizes and the inclusion of multiple aphid lines representing each of the two *Buchnera* alleles. Nonetheless, aphids with the alternative *Buchnera* alleles showed dramatic differences in performance when subjected to heat as juveniles. In fact, most aphids containing *Buchnera* with the short allele failed to reproduce at all following heat stress, whereas almost all aphids containing *Buchnera* with the long allele did reproduce (Table 2, χ^2^ = 10.80, *df* = 1, *p* ≤ 0.0025). This effect of the *Buchnera* allele was evident for different origins of the short allele and whether or not aphids were infected with *Candidatus* Serratia symbiotica, which previously was shown to impact heat tolerance [6,13]. Because aphids bearing *Buchnera* with the short allele rarely reproduced following heat treatment, the other measures of performance could not be compared between the short and long lines for this treatment.

**Table 2:**
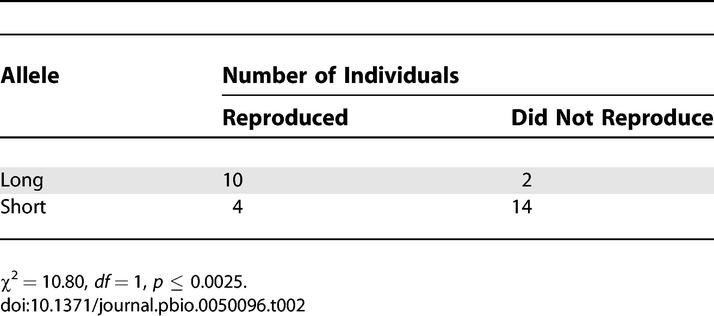
Reproductive Ability Following Heat Stress of Aphids Derived from the Same Clonal Lineage, 5A, with Alternative *Buchnera ibpA* Heat-Shock Promoter Alleles

**Figure 4:**
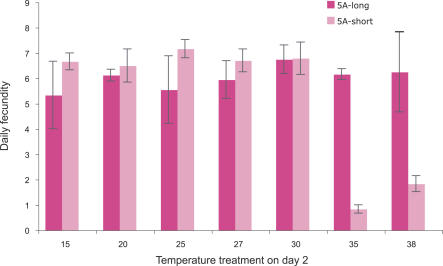
Effect of Different Temperature Treatments on Day 2 on Fecundity as Adults, for Aphid Lines with *Buchnera* with Different *ibpA* Promoter Alleles Bars show means ± standard errors.

In a second set of experiments designed to have greater power to detect effects of the *Buchnera* polymorphism on host performance, we examined consequences for adult fecundity of a 4-h exposure on day 2 to a variety of temperatures, ranging from 15–38 °C. In these experiments, we compared two 5A-derived lines: one containing *Buchnera* with the short allele and one containing *Buchnera* with the long allele. Both were free of facultative symbionts. Only under the severe heat-stress treatments (35 °C and 38 °C) did a highly significant difference emerge: as in the previous experiment, heat stress within 48 h of birth results in almost complete reproductive failure of aphids containing short-allele *Buchnera* (Figure 4).

**Figure 5:**
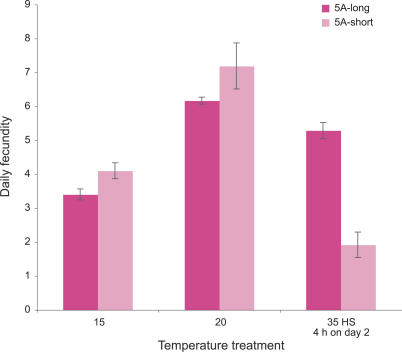
Reproductive Rates under Different Temperature Conditions of Aphid Lines with *Buchnera* with Different *ibpA* Promoter Alleles Number of nymphs per day for the first 6 d of reproduction for 5A females with *Buchnera* bearing the long and short alleles (5A^−^R^LONG^and 5A^SHORT^). Bars show means ± standard errors. Temperature conditions were constant 15 °C, constant 20 °C, and 20 °C with 4 h at 35 °C on day 2 following birth.

To determine whether aphids with short-allele *Buchnera* have an advantage at constant low temperatures, we conducted an additional experiment comparing the same pair of 5A lines reared under three thermal treatments: 15 °C, 20 °C, and 20 °C with 4 h of 35 °C at the beginning of day 2. Aphids with the short-allele *Buchnera* had a significant performance advantage at both 15 °C and 20 °C. At 15 °C, time from birth to adulthood did not differ significantly, but time between adulthood and first reproduction did differ significantly. Most aphids with long-allele *Buchnera* did not reproduce for more than 48 h after attaining adulthood, and most of the aphids with short-allele *Buchnera* took less than 48 h (Figure 5A, Fisher exact test, *p* < 0.000001). At both 15 °C and 20 °C, rate of progeny production was higher for aphids containing *Buchnera* with the short allele (Figure 5; 15 °C: Student *t,* two-tailed, *t* = 2.51, *df* = 32, *p* < 0.02; 20 °C: *t* = 2.34, *df* = 32, *p* < 0.03). As in the previous experiment, under the treatment involving heat on day 2, aphids containing *Buchnera* with the short allele performed much worse. Even after removing the ones that failed to reproduce at all, the rate of reproduction was far lower than for aphids containing *Buchnera* with the long allele (Figure 5, *t* = 5.48, *df* = 33, *p* < 0.0001). Thus, under constant cool or moderate conditions, aphids with short-allele *Buchnera* have significantly higher reproductive rates, but when exposed to heat as juveniles, aphids with short-allele *Buchnera* reproduce at much lower rates.

We conclude that the alternative symbiont genotypes are beneficial to hosts under different thermal environments. The long-allele *Buchnera* confer an advantage to hosts that experience heat stress as a juvenile, whereas the short-allele *Buchnera* enable hosts to perform better under constant cool conditions. Both sets of conditions are within the range of environments that are relevant for A. pisum depending on geographic location, weather, and season.

### Effect of *ibpA* Promoter Allele on *Buchnera* Numbers Following Heat Stress

**Figure 6:**
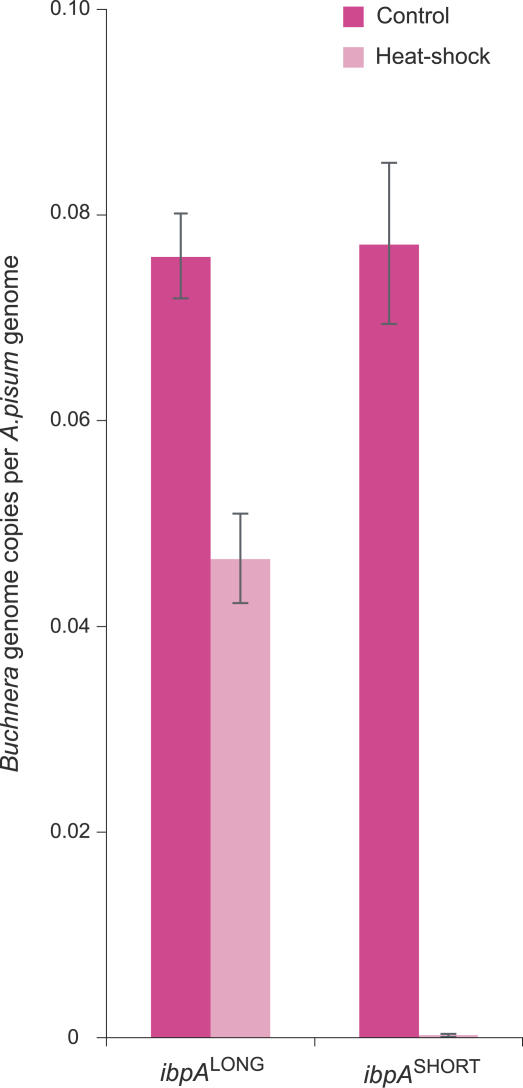
Effect of Heat Stress on *Buchnera* Densities in Aphids Bearing *Buchnera* with Different *ibpA* Promoter Alleles Estimates of *Buchnera* and aphid genome numbers are based on qPCR assays of genomic DNA determining numbers of copies of single-copy *Buchnera* genes relative to a single-copy aphid gene. Heat treatment was 4 h at 35 °C on day 2 following birth, and measures were taken within 24 h of attaining adulthood. Bars show means ± standard errors.

To determine whether the *ibpA* response to heat stress affects maintenance of *Buchnera* cell populations within hosts, we compared titers of *Buchnera* in aphids with the alternative *Buchnera* types but otherwise having similar genetic background; treatments consisted of heat stress of day 2 and control conditions (constant 20 °C). For the control treatment, *Buchnera* genome copy number did not differ between lines bearing the two alleles (Figure 6, *W* = 72.00, *p* = 0.72). However, for the heat-treated aphids, we found highly significant differences (Figure 6, *W* = 36.00 *p* < 0.0001); those with *Buchnera* bearing the short allele had almost no *Buchnera* (mean = 9.3 × 10^2^). These levels were only slightly above the limit of detection and less than 0.1% of the numbers found in controls of the same aphid genotypes. In contrast, the aphids bearing *Buchnera* with the long allele retained substantial *Buchnera* populations (mean = 5.2 × 10^5^), though they were reduced to about 30% of those of controls. In these same aphids, the heat exposure as juveniles reduced the adult weight of individuals with short-allele *Buchnera* to less than 40% of controls (averages of 1.05 mg versus 2.74 mg); heat reduced the adult weight of the long-allele individuals slightly, to 80% of that of controls (2.38 mg versus 2.97 mg).

### Conservation of Spacer of *ibpA* Promoter across *Buchnera* Lineages

The *ibpA* promoter spacer contained a homopolymeric run of adenines in the sequenced genomes of *Buchnera* of both A. pisum and *S. graminum,* despite the fact that most neutral regions are highly divergent between these two lineages [3]. To determine whether this homopolymeric tract is conserved across *Buchnera* more generally, we sequenced the *hslU-ibpA* spacer in four additional aphid species, representing two aphid tribes (Macrosiphini and Aphidini). All species had a homopolymeric run of adenine nucleotides in the spacer of the promoter, resulting in low sequence divergence in the spacer relative to that in the non-coding sequences flanking the promoter (Figure S1). Regions flanking the promoter were highly divergent and not readily aligned, consistent with extensive sequence divergence at neutral sites between *Buchnera* from different aphid lineages. For example, the divergence of *Buchnera* of S. graminum and *Buchnera* of A. pisum is close to one substitution per neutral position, and most intergenic spacers cannot be aligned between these lineages [3]. In contrast, the spacer in the *ibpA* promoter differs in only one of 13 positions (Figures 1B and S1). The presence of the homopolymeric run in all sampled species is evidence of conservation of this feature by purifying selection. It also suggests that similar mutations affecting heat tolerance are occurring in *Buchnera* of other aphids.

## Discussion

We show that a single-base deletion in the obligate symbiont *Buchnera* causes symbiont numbers to plummet and results in reproductive failure or very low fecundity in aphid hosts that are subjected to short heat exposure as juveniles. Many studies show that aphids with few or no *Buchnera* experience severe reductions in fecundity [5,12,13], and thus, the deleterious effect of the short allele on aphid fitness probably reflects *Buchnera* cellular death and/or failure to replicate within hosts as they grow. Further, we have shown that this mutation is beneficial to host fitness under constant cool conditions, that it occurs frequently, that it can increase in frequency under some conditions (based on its fixation in laboratory lines), and that it is moderately frequent in field collections. Together these observations strongly suggest that this allele is sometimes favored by selection.

Although some aphids in this study are infected with additional, facultative symbionts that can affect tolerance to heat [6,14], our comparisons controlled for differences in facultative symbiont infections. They also controlled for differences in aphid genotype, because mutations occurred independently in individuals recently derived from a single female. Thus, we conclude that this mutation in an obligate symbiont has an overwhelming effect on reproductive performance in the face of heat stress.

Although we have not identified the precise mode through which *ibpA* expression benefits aphids during heat stress and lowers performance during cool conditions, studies on the role of *ibpA* in E. coli and other bacteria provide potential clues. The small heat-shock proteins IbpA and IbpB bind with a broad range of protein species, preventing misfolded polypeptides from becoming irreversibly aggregated and then transferring them to ATP-dependent chaperones, such as DnaK (= Hsp70). Because binding with Ibp does not require ATP, interaction with this small heat-shock protein is hypothesized to arrest aggregation of misfolded proteins during periods of cellular ATP depletion so that they can be rescued later by ATP-requiring chaperones [15–18]. High expression of *ibpB* (a homolog of *ibpA*) appears to slow cell growth under non-stress conditions, an effect hypothesized to result from indiscriminant binding of the IbpB protein with cellular constituents [17]. In the case of *Buchnera,* the simplest hypothesis would be that IbpA helps prevent irreversible protein aggregation under heat stress and reduces cell functionality by binding with protein substrates at lower temperatures. This explanation is consistent with the elimination of short-allele *Buchnera* following heat stress. The effect of the short allele in reducing *ibpA* expression even under cool conditions (Figure 2C) strongly suggests that elevated levels of IbpA reduce *Buchnera* functionality at lower temperatures, resulting in lower growth and fecundity of aphids bearing *Buchnera* with the long allele. Deleterious effects of increased expression of a heat-shock protein have also been reported in eukaryotic systems (e.g., see [19,20]).

Remarkably, the mutation to the short allele occurred and became fixed twice in the laboratory. All laboratory stocks, including those in which the short allele evolved, have been kept at constant 20 °C for intervals ranging up to 6 y prior to this study. The cool and stable thermal environment likely facilitated spread of the mutation. If the allele is completely neutral under the laboratory conditions, the mutation rate from long to short, as estimated in the 5A background, is approximately once per 100 aphid generations (based on a mean generation time of 12 d and 2,400 d of culture), or approximately once per 800 *Buchnera* divisions [21], that is, approximately 1 × 10^−3^ for this particular mutation. This is a remarkably high estimate, even considering that indels are frequent in homopolymeric runs (e.g., see [22]) and that spontaneous mutation rates in *Buchnera* and other symbionts are probably higher than in other bacteria (e.g., see [23]), possibly due to the loss of DNA repair genes [1], some of which affect indel rates in homopolymeric stretches [24]. The rate approximates the upper estimates for the genome-wide mutation rate in *Buchnera* and other bacteria, and is about 1,000-fold higher than per-site estimates of mutation [22]. Furthermore a genome-wide screen of *Buchnera* mutations in sublines of 5A revealed only a single other mutation fixed in one subline and none affecting the sublines used in this study (unpublished data).

The fixations of the short *ibpA* allele thus suggest a selective advantage under the laboratory conditions, an observation that is consistent with the experimental results showing that hosts containing *Buchnera* with the short allele have a significant advantage at 15 °C and at 20 °C (Figure 5).

The frequency of the short allele in some populations (up to 21%; Table 1) also is consistent with an occasional fitness advantage. Under the assumption that the short allele is consistently deleterious in the field and is maintained only by recurrent mutation, its equilibrium frequency would be approximately equal to the mutation rate divided by the selective disadvantage [25]. Under this scenario of mutation-selection balance, a frequency of 10% or more would imply an implausibly high mutation rate for an allele with a substantial deleterious effect. For example, for an average reduction in aphid fitness of 0.1, the implied mutation rate would be greater than 1 × 10^−2^ per aphid generation (and proportionally higher for a stronger selective disadvantage). This rate again appears to be improbably high, suggesting that recurrent mutation alone cannot account for the observed allele frequency, and providing evidence that the short allele is beneficial under some field conditions. We hypothesize that aphids with *Buchnera* bearing this allele sometimes experience positive selection depending on geographical location and weather, and that this selection together with recurrent mutation maintains the polymorphism.

The support for a fitness advantage of the short allele under some conditions leads us to speculate that the homopolymeric run of adenines in the *ibpA* heat-shock promoter potentially has been conserved for its evolvability, allowing host lineages to undergo reversible microevolution in response to changing thermal environments. This possibility is consistent with the finding that the homopolymeric run within this promoter is conserved in genomes of *Buchnera* from different aphid host species (Figure S1).

### Conclusions

Thermal tolerance plays a major role in limiting the geographic and altitudinal distributions of species and of particular genotypes within species (e.g., see [26]). Many cases of genotypic variation in heat tolerance are known, and distributions of alleles in invertebrate populations have the potential to serve as indicators of changing climatic conditions [27,28]. In view of the dependence of many invertebrates on vertically transmitted microbial symbionts with high mutation rates, it is likely that microevolution in symbionts frequently impacts host tolerances. The case of the *ibpA* promoter in *Buchnera* provides a clear example of such an impact. This example illustrates that the biological complexity imposed by obligate symbiotic associations has significant consequences for how hosts are affected by changing environmental conditions.

## Materials and Methods

### Laboratory lines of *A. pisum.*

Lines of A. pisum used in this study were maintained in a walk-in plant growth room on fava beans, in cages constructed from Plexiglass and fine mesh. Each cage contained four pots, each with three plants. All clones were maintained in continuous parthenogenetic culture on fava bean at 20 °C and long-day conditions (16 h of light to 8 h of darkness). Numerous protocols were implemented to prevent contamination of cultures by aphids escaping cages and entering other colonies. In addition, the clonal integrity of laboratory lines was verified at regular intervals from the time of their isolation using amplified fragment length polymorphisms that are diagnostic for each clone and that are described in Oliver et al. [10]. In all, five laboratory lines were investigated or screened in this study. Every line is descended from a single parthenogenetic female collected directly from the field: 7-2-1, 9-2-1, and 7A were collected by J. Russell from alfalfa in August 2001 in Cayuga County, New York; TUC was collected by N. Moran in May 1999 from Vicia fava in Tucson, Arizona, and line 5A was collected by N. Moran in June 1999 from a wild legume in Madison, Wisconsin. Line 5A was used as a source of sublines, each established with a single female taken from the 5A colony at different points, and sometimes infected with a facultative symbiont [10]. During long-term maintenance of cultures, aphids were allowed to increase to high densities in the cages and then were transferred to fresh plants by inoculating with a small number (10–20) of individuals from the culture. At each culture change (approximately once per month), samples were removed and reserved at −80 °C.

### Microarray heat-shock experiments.

Three lines of A. pisum (5A, 5AR, and TUC) were used in microarray heat-shock experiments. We subjected two biological replicates of each aphid line to a 3-h 35.5 °C ± 0.5 °C heat-shock treatment. Comparing these samples to non–heat-shocked control lines, microarray hybridization experiments on each biological replicate (two slides per biological replicate [with dye switched between treatments between slides] times two biological replicates per aphid line times three aphid lines equals 12 slides total). Microarray construction, RNA extraction, microarray hybridization, and statistical analyses are described in Wilson et al. [9] for the A. pisum/*Buchnera* dual-genome microarray.

### Verification of dimorphic heat-shock expression pattern with RT-qPCR.

We verified microarray results with RT-qPCR from cDNA for four of the *Buchnera* genes that appeared to be up-regulated under heat-shock conditions *(dnaK, ibpA, mopA,* and *ψyjeA)* and two *Buchnera* genes that did not show a heat-shock response *(argG* and *cysG)* using the primers and methods described in Wilson et al. [9]. In addition to confirming gene expression in both biological replicates of the TUC aphid line and one biological replicate of each of the 5A and 5AR lines examined by microarray hybridization, we analyzed *Buchnera ibpA* expression in two more aphid lines: 7-2-1 and 9-2-1. This experiment was aimed at showing that the *ibpA* spacer polymorphism had an overriding effect on *ibpA* response to heat shock, regardless of other differences in *Buchnera* and aphid genetic background.

### Determination of effects of different temperatures on *ibpA* expression for *Buchnera* with short and long *ibpA* promoter alleles.

We performed experiments with 5A^SHORT^, 5A^−^R^LONG^, and TUC to examine the effect of different temperature treatments on *ibpA* expression for the short and long *Buchnera* alleles. Each line was grown in three sublines for at least two generations at continuous 20 °C, as outlined above, and adults from these sublines were used to produce cohorts of newborn juveniles. On day 2 following birth, aphids were subjected to 4-h treatments of 20 °C, 25 °C, 27 °C, 30 °C, 35 °C, and 38 °C, by moving their host plants from a 20 °C growth chamber to a chamber at the specified temperature. The 20 °C treatment consisted of continuous rearing at 20 °C. At the end of the 4 h, aphids were immediately frozen in liquid nitrogen for determination of transcript levels for *ibpA* and *cysG* using RT-qPCR, with three aphids assayed individually for each combination of aphid line and treatment (one aphid per subline). Methods were the same as those outlined above for determining transcript levels. In addition, we compared *ibpA* and *cysG* levels for 5A^SHORT^ and 5A^−^R^LONG^ reared at continuous 15 °C.

### Discovery of the *ibpA* promoter polymorphism in *Buchnera* of *A. pisum.*

We designed the following PCR primers to amplify the intergenic region upstream of the *Buchnera* of *A. pisum ibpA* start site: ibpAf: 5′ CATCTAATGAAGATCTTGGTCGTTT 3′, ibpAr: 5′ TTTGGTATAAATGAGAGAGAACGAT 3′. PCR reactions were carried out in 25-μl reaction volumes containing 50 mM KCl, 10 mM Tris-HCl, 1.5 mM Mg^2+^, 2.5 mM each of dNTP, 0.4 units of Eppendorf *Taq* DNA polymerase, and approximately 10 ng of DNA. We used a touchdown PCR reaction with profile of 94 °C for 2 min, followed by two cycles of 94 °C for 15 sec, (62 °C −1 °C) for 30 sec, 72 °C for 45 sec, three cycles of 94 °C for 15 sec, (61 °C −2 °C) for 30 sec, 72 °C for 45 sec, and 30 cycles of 94 °C for 15 sec, 55 °C for 30 sec, 72 °C for 45 sec; final extension at 72 °C for 6 min. PCR products were sequenced with the PCR primers on an ABI 3700 by the Genomic Analysis and Technology Core facility at the University of Arizona.

### Population and species variation in the *Buchnera ibpA* promoter.

We screened individuals from 88 field-collected A. pisum lines for DNA sequence variation in the *ibpA* σ32 binding site. All lines were collected in the United States: near Madison, Wisconsin; Cayuga County and Tomkins County, New York; Logan Utah; and Tucson Arizona. In addition, we examined multiple individuals from 12 laboratory lines, each derived from a single field-collected female. Laboratory lines included six sublines derived from line 5A between 1999 and 2006. These sublines had been isolated as single females from the main 5A line and, in some cases, transfected with different aphid secondary bacterial symbionts [13]. For laboratory lines, including sublines of 5A, we screened the *ibpA* promoter for samples that had been frozen periodically from the time the line was established from a field-collected female. Methods for PCR and sequencing were the same as described in the previous section.

We also determined sequence for the *hslU-ibpA* spacer for *Buchnera* of other aphid species, including *Aphis gossypii, Uroleucon ambrosiae,* and *Sitobion avenae,* all collected from Tucson, Arizona, and *Myzus persicae* collected from Windsor, Connecticut, from tobacco by Jim Lamondia. We used a generic pair of primers that perfectly match the published genome sequences of the *Buchnera* from A. pisum and *S. graminum,* located in the coding regions of the genes flanking the spacer: ibpAgenf 5′ GCTTGGAAAGTTAATGAATCTAT 3′ and ibpAgenr 5′ TTTTATCAATTTGATTGAATCTATTTG 3′. We used the same primers for PCR amplification and sequencing, with PCR and sequencing methods as outlined above.

### Aphid performance experiments.

In an initial experiment aimed at examining the effect of the *Buchnera* mutation on host performance, we used four aphid lines: 5A^+^R^LONG^, 5A^SHORT^, 5A^−^R^LONG^_,_ and 5A^−^R^SHORT^. These lines were all derived as clones descending from a single parthenogenetic female 6 y earlier (Figure 3). The superscript SHORT indicates that the line has a 12-bp spacer in the *Buchnera ibpA* σ32 heat-shock promoter binding site, and the superscript LONG indicates that the line has a 13-bp spacer (Figure 1B). When first established from a field collection in June 1999, line 5A bore the long allele and no secondary endosymbiont. In September of 2000, a subline of 5A^LONG^ was transfected with the facultative bacterial endosymbiont, *Candidatus* Serratia symbiotica (previously known as the R-type) to generate the 5A^+^R^LONG^ line. In January 2001 (or within the two preceding months), the *Buchnera* of 5A^LONG^ acquired a mutation in the binding site of the σ32 heat-shock promoter; the mutation became fixed in this line, which became 5A^SHORT^. In October 2005, we discovered that 5A^+^R^LONG^ was polymorphic for both *Buchnera* alleles, with the long allele near fixation. We isolated two lines, representing each allele, and refer to them as 5A^+^R^LONG^ and 5A^+^R^SHORT^. In October and November 2005, the *Candidatus* Serratia symbiotica infection was lost from 5A^+^R^SHORT^ and was removed using heat (constant 30 °C for 5 d at 14-h light:10-h dark) from a subline isolated from 5A^+^R^LONG^. We refer to these cured lines as 5A^−^R^SHORT^ and 5A^−^R^LONG^.

Two sublines of each aphid line used in the experiments were established on fava bean seedlings and maintained for three generations prior to the start of the experiment, to control for possible maternal effects reflecting recent rearing conditions. For each subline, a control and a treatment plant was established with ten fourth instar aphids per plant. These aphids became adult and began to reproduce; they were removed from plants and preserved for DNA work 24 h after giving birth to their first progeny. Under the heat treatment, 1–2-d-old progeny were heat shocked at 35.5 °C ± 0.5 °C for a 4-h period. Control individuals were held at constant 20 °C. Following heat shock, cages were returned to 20 °C. When test aphids were fourth instars, three individuals were chosen at random from the control and heat treatments, and transferred to new plants, with one aphid per plant. For each of these individuals, we recorded the following: (1) age at first reproduction, (2) number of progeny over first 7 d of reproduction, (3) total weight (mg) of progeny, and (4) maternal weight (mg) after 7 d of reproduction.

The distributions of all aphid fitness measures were non-normal, even after attempts to transform the data. Therefore, we analyzed the performance experiment data using non-parametric Wilcoxon tests implemented in SAS 9.1 (proc npar1way). We computed exact probability statistics based on the Wilcoxon scores for age at first reproduction, number of progeny, progeny weight data, and maternal weight for the aphid lines held under control conditions, to test for the effect of promoter allele on aphid performance The heat-treatment data could not be analyzed in the same way as the control data because most heat-treated aphids failed to reproduce. Hence, we scored lines as having reproduced or not reproduced following heat treatment and tested for the effect of promoter allele using a *χ2* test.

In a subsequent experiment, we explored the effect of the *Buchnera* polymorphism on host performance following exposure to a range of temperatures, using 5A^SHORT^ and 5A^−^R^LONG^ (both lacking secondary symbionts and having the same genotype other than the *Buchnera* polymorphism). To control for colony and maternal effects, each of the two lines was grown as four sublines for two generations; adults removed from these sublines were used to initiate experiments, with one experimental colony per subline (giving four independent measures for each line at each treatment). These adult females were allowed to deposit nymphs on a new plant overnight, then adults were removed. Nymphs were left for 24 h, then subjected to the treatment temperature, if any, for 4 h and returned to constant 20 °C. These nymphs were allowed to develop and reproduce. Day of adulthood and daily fecundity were recorded, with progeny removed daily for 6 d. Individuals that failed to reproduce at all were excluded from fecundity measures. A total of 50 individual measures of daily fecundities were obtained (seven treatments times four sublines per line times two lines minus six failed).

In a third performance experiment, we addressed whether the *Buchnera* polymorphism affected aphid performance at a constant moderate or cool temperature versus moderate temperature with brief heat stress as a juvenile. Test aphids came from the same four sublines for both 5A^−^R^LONG^ and 5A^SHORT^; prior to the experiment, sublines were grown independently at constant 20 °C for at least four generations. Adults from these sublines were used to generate nymphs on the test plants, with time of birth recorded within a 4-h interval and with one to four nymphs per mother. For the 20 °C treatment, plants were kept at the same temperature following removal of adults. For the 15 °C treatment, plants were transferred to a constant 15 °C chamber as soon as adults were removed. For the heat-stress treatment, adults were removed, progeny were allowed to develop for 24 h and were then subjected to 4 h at 35 °C and returned to 20 °C to develop. Aphids were checked daily, with date of adulthood, date of first reproduction, and daily progeny production recorded until day 6. At both 15 °C and 20 °C, a minority of individuals developed into winged morphs; these were excluded from analyses because winged forms are known to have different developmental time and patterns of fecundity. Student *t*-tests were used to analyze average daily nymph production, and Fisher exact tests were used to analyze time to adulthood and time from adulthood to first reproduction, with time recorded within categories of 24-h intervals.

### Effect of *ibpA* promoter allele on *Buchnera* numbers following heat shock.

Two-day-old aphids from aphid lines 5A^−^R^SHORT^, 5A^SHORT^, 5A^+^R^LONG^, and 5A^−^R^LONG^ were heat shocked following the treatment used in the microarray experiments. Control aphids were held at constant 20 °C. When control and heat-shocked aphids reached day 1–2 of adulthood (prior to first reproduction), we collected them into liquid nitrogen and stored them for subsequent analysis of absolute *Buchnera* chromosome copy number using qPCR and primers designed to the single-copy *Buchnera* gene *(dnaK)* and a single-copy aphid nuclear gene *(EF 1-alpha)*. DNA was extracted from single aphids using Qiagen DNeasy DNA extraction columns with the optional RNase A step for the removal of RNA (Qiagen, Valencia, California, United States). We used PCR primers BHS70F2 5' ATGGGTAAAATTATTGGTATTG 3' and BHS70R2 5' ATAGCTTGACGTTTAGCAGG 3' to amplify the single-copy *Buchnera* gene, *dnaK,* and primers ApEF1-alpha 107F 5' CTGATTGTGCCGTGCTTATTG 3' and ApEF1-alpha 246R 5' TATGGTGGTTCAGTAGAGTCC 3' to amplify the single-copy aphid gene, *elongation factor 1-α (EF 1-alpha)*. We followed the standard quantitative touchdown PCR protocol detailed in Moran et al. [29]. Quantification of *Buchnera* genome copy number followed the methods of Plague et al. [30].

We computed the *Buchnera* densities by dividing relative *Buchnera* gene copy number by relative copy number of the single-copy aphid gene, to estimate the number of *Buchnera* genomes per aphid genome. Using a Wilcoxon signed rank test implemented in SAS 9.1 (proc npar1way), we compared measures of *Buchnera* density for the alternative alleles in control replicates (Wilcoxon test statistics, means ± standard errors for long and short, respectively: *W* = 72.00, *p* = 0.72; 0.076 ± 0.004, 0.077 ± 0.008) and also the difference in *Buchnera* density between control and treatment replicates for each allele (*W* = 97.0, *p* = 0.0011; 0.029 ± 0.007, 0.077 ± 0.008). We also tested for an effect of secondary symbiont infection on *Buchnera* densities in the long lines and found no significant differences between the responses of infected and uninfected long lines (*W* = 11.0, *p* = 0.06, mean for aphids with long-allele *Buchnera* and infected with *Candidatus* Serratia symbiotica: 0.039 ± 0.004, mean for aphids with long-allele *Buchnera* and uninfected with secondary: 0.054 ± 0.006).

## Supporting Information

Figure S1 *ibpA* Heat-Shock Promoter and Intergenic Flanking Sequence from *Buchnera* of Two Aphid Tribes: Aphidini and Macrosiphini The black line indicates the σ32 promoter (note the low divergence of the six species across these 28 bp). Position 1 is the last position of the stop codon of the upstream gene, *hslU*. The intergenic region outside the σ32 binding site shows no detectable homology and was not aligned.
(603 KB PNG)

### Accession Numbers

The GenBank (http://www.ncbi.nlm.nih.gov/Genbank) accession numbers for the genetic material discussed in this paper are as follows: alleles from A. pisum lines (DQ889712–DQ889714 and NC002528); Buchnera aphidicola genome (NC002528); and the *hslU-ibpA* spacer from Aphis gossypii (DQ897656), Myzus persicae (DQ897659), Schizaphis graminum (NC004061), Sitobion avenae (DQ897659), and *Uroleucon ambrosiae* (DQ897657).

## Abbreviations

bp: base pair
qPCR: quantitative PCR
RT-qPCR: reverse transcriptase quantitative PCR

## References

[pbio-0050096-b001] Shigenobu S, Watanabe H, Hattori M, Sakaki Y, Ishikawa H (2000). Genome sequence of the endocellular bacterial symbiont of aphids Buchnera sp. APS. Nature.

[pbio-0050096-b002] Baumann P (2005). Biology of bacteriocyte-associated endosymbionts of plant sap-sucking insects. Annu Rev Microbiol.

[pbio-0050096-b003] Tamas I, Klasson L, Canback B, Naslund AK, Eriksson AS (2002). 50 Million years of genomic stasis in endosymbiotic bacteria. Science.

[pbio-0050096-b004] Moran NA, Munson MA, Baumann P, Ishikawa H (1993). A molecular clock in endosymbiotic bacteria is calibrated using the insect hosts. Proc Roy Soc Lond B Biol Sci.

[pbio-0050096-b005] Ohtaka C, Ishikawa H (1991). Effects of heat treatment on the symbiotic system of an aphid mycetocyte. Symbiosis.

[pbio-0050096-b006] Montllor CB, Maxmen A, Purcell AH (2002). Facultative bacterial endosymbionts benefit pea aphids Acyrthosiphon pisum under heat stress. Ecol Entomol.

[pbio-0050096-b007] Feder ME, Hofmann GE (1999). Heat-shock proteins, molecular chaperones, and the stress response: Evolutionary and ecological physiology. Annu Rev Physiol.

[pbio-0050096-b008] Wilcox JL, Dunbar HE, Wolfinger RD, Moran NA (2003). Consequences of reductive evolution for gene expression in an obligate endosymbiont. Mol Microbiol.

[pbio-0050096-b009] Wilson ACC, Dunbar HE, Davis GK, Hunter WB, Stern DL (2006). A dual-genome microarray for the pea aphid, *Acyrthosiphon pisum,* and its obligate symbiont, Buchnera aphidicola. BMC Genomics.

[pbio-0050096-b010] Oliver KM, Russell JA, Moran NA, Hunter MS (2003). Facultative bacterial symbionts in aphids confer resistance to parasitic wasps. Proc Natl Acad Sci U S A.

[pbio-0050096-b011] Wang Y, deHaseth PL (2003). Sigma 32-dependent promoter activity in vivo: Sequence determinants of the groE promoter. J Bacteriol.

[pbio-0050096-b012] Sasaki T, Hayashi H, Ishikawa H (1991). Growth and reproduction of the symbiotic and aposymbiotic pea aphids, Acyrthosiphon pisum maintained on artificial diets. J Insect Physiol.

[pbio-0050096-b013] Douglas AE, Prosser WA (1992). Synthesis of the essential amino acid tryptophan in the peas aphids *(Acyrthosiphon pisum)* symbiosis. J Insect Physiol.

[pbio-0050096-b014] Russell J, Moran NA (2006). Costs and benefits of symbiont infection in aphids: Variation among symbionts and across temperatures. Proc Roy Soc Lond B Biol Sci.

[pbio-0050096-b015] LeThanh H, Neubauer P, Hoffmann F (2005). The small heat-shock proteins IbpA and IbpB reduce the stress load of recombinant Escherichia coli and delay degradation of inclusion bodies. Microb Cell Fact.

[pbio-0050096-b016] Mogk A, Deuerling E, Vorderwulbecke S, Vierling E, Bukau B (2003). Small heat shock proteins, ClpB and the DnaK system form a functional triade in reversing protein aggregation. Mol Microbiol.

[pbio-0050096-b017] Kuczynska-Wisnik D, Kcdzierska S, Matuszewska E, Lund P, Taylor A (2002). The Escherichia coli small heat-shock proteins IbpA and IbpB prevent the aggregation of endogenous proteins denatured in vivo during extreme heat shock. Microbiology.

[pbio-0050096-b018] Kitagawa M, Miyakawa M, Matsumura Y, Tsuchido T (2002). Escherichia coli small heat shock proteins, IbpA and IbpB, protect enzymes from inactivation by heat and oxidants. Eur J Biochem.

[pbio-0050096-b019] Krebs RA, Feder ME (1997). Deleterious consequences of Hsp70 overexpression in Drosophila melanogaster larvae. Cell Stress Chaperones.

[pbio-0050096-b020] Williams KD, Helin AB, Posluszny J, Roberts SP, Feder ME (2003). Effect of heat shock, pretreatment and hsp70 copy number on wing development in Drosophila melanogaster. Mol Ecol.

[pbio-0050096-b021] Mira A, Moran NA (2002). Estimating population size and transmission bottlenecks in maternally transmitted endosymbiotic bacteria. Microb Ecol.

[pbio-0050096-b022] Denver DR, Morris K, Kewalramani A, Harris KE, Chow A (2004). Abundance, distribution, and mutation rates of homopolymeric nucleotide runs in the genome of Caenorhabditis elegans. J Mol Evol.

[pbio-0050096-b023] Ochman H, Elwyn S, Moran NA (1999). Calibrating bacterial evolution. Proc Natl Acad Sci U S A.

[pbio-0050096-b024] Denver DR, Feinberg S, Estes S, Thomas WK, Lynch M (2005). Mutation rates, spectra and hotspots in mismatch repair-deficient Caenorhabditis elegans. Genetics.

[pbio-0050096-b025] Crow JF (1986). Basic concepts in population, quantitative, and evolutionary genetics.

[pbio-0050096-b026] Balanya J, Oller JM, Huey RB, Gilchrist GW, Serra L (2006). Global genetic change tracks global climate warming in Drosophila subobscura. Science.

[pbio-0050096-b027] Umina PA, Weeks AR, Kearney MR, McCKechnie SW, Hoffmann AA (2005). A rapid shift in a classic clinal pattern in *Drosophila* reflecting climate change. Science.

[pbio-0050096-b028] Bradshaw WE, Holzapfel CM (2006). Evolutionary response to rapid climate change. Science.

[pbio-0050096-b029] Moran NA, Degnan PH, Santos SR, Dunbar HE, Ochman H (2005). The players in a mutualistic symbiosis: Insects, bacteria, viruses, and virulence genes. Proc Natl Acad Sci U S A.

[pbio-0050096-b030] Plague GP, Dale C, Moran NA (2003). Low and homogeneous copy number of plasmid-borne symbiont genes affecting host nutrition in Buchnera aphidicola of the aphid Uroleucon ambrosiae. Mol Ecol.

